# Primary Tumor Resection for Metastatic Colorectal, Gastric and Pancreatic Cancer Patients: In Search of Scientific Evidence to Inform Clinical Practice

**DOI:** 10.3390/cancers15030900

**Published:** 2023-01-31

**Authors:** Valentina Fanotto, Francesca Salani, Caterina Vivaldi, Mario Scartozzi, Dario Ribero, Marco Puzzoni, Francesco Montagnani, Francesco Leone, Enrico Vasile, Maria Bencivenga, Giovanni De Manzoni, Debora Basile, Lorenzo Fornaro, Gianluca Masi, Giuseppe Aprile

**Affiliations:** 1 Department of Oncology, Academic Hospital of Udine, Azienda Sanitaria Universitaria Friuli Centrale (ASUFC), Piazzale Santa Maria della Misericordia, 33100 Udine, Italy; 2 Unit of Oncology 2, Azienda Ospedaliero-Universitaria Pisana, 56126 Pisa, Italy; 3 Institute of Interdisciplinary Research “Health Science”, Scuola Superiore Sant’Anna, Piazza Martiri della Libertà 33, 56124 Pisa, Italy; 4 Department of Translational Research and New Technologies in Medicine and Surgery, University of Pisa, 56126 Pisa, Italy; 5 Unit of Medical Oncology, University Hospital, University of Cagliari, 09124 Cagliari, Italy; 6 Division of General and Oncologic Surgery Multimedica, A.O. Santa Croce e Carle, 12100 Cuneo, Italy; 7 Department of Oncology, Azienda Sanitaria Locale di Biella, 13900 Ponderano, Italy; 8 General and Upper GI Surgery Division, Verona University (VR), 37134 Verona, Italy; 9 Department of Oncology, San Bortolo General Hospital, ULSS 8 Berica-Vicenza, 36100 Vicenza, Italy

**Keywords:** colorectal cancer, pancreatic adenocarcinoma, gastric adenocarcinoma, surgery, stage IV, primary tumor resection

## Abstract

**Simple Summary:**

Oncology practice in gastrointestinal tumors is moving toward therapeutic algorithms comprising multiple systemic options integrated with loco-regional strategies, such as surgery. This paradigm holds true for metastatic colorectal cancer, as well as upper gastrointestinal neoplasms, where the role of the resection of the primary tumor, with or without the administration of systemic therapies and metastasectomy, has been a matter of debate. In our review paper, we discuss the available randomized and retrospective evidence supporting surgery in the metastatic setting of colorectal, gastric and pancreatic cancers, with the aim to grant the clinicians with an up-to-date state of the art on this subject.

**Abstract:**

The management of the primary tumor in metastatic colorectal, gastric and pancreatic cancer patients may be challenging. Indeed, primary tumor progression could be associated with severe symptoms, compromising the quality of life and the feasibility of effective systemic therapy, and might result in life-threatening complications. While retrospective series have suggested that surgery on the primary tumor may confer a survival advantage even in asymptomatic patients, randomized trials seem not to definitively support this hypothesis. We discuss the evidence for and against primary tumor resection for patients with metastatic gastrointestinal (colorectal, gastric and pancreatic) cancers treated with systemic therapies and put in context the pros and cons of the onco-surgical approach in the time of precision oncology. We also evaluate current ongoing trials on this topic, anticipating how these will influence both research and everyday practice.

## 1. Introduction

Colorectal (CRC), gastric (GC) and pancreatic cancers (PC) are among the major causes of cancer death worldwide [1]. While effective screening procedures enhanced the chances of cure for CRC [2], GC and PC are often diagnosed at advanced stages, when the main treatment goal is palliation. Moreover, even when an apparently radical surgery is possible, distant recurrence is a major unresolved issue [3], thus establishing systemic therapy as the mainstay of treatment. In the last years, along with the effort of improving patients’ overall survival (OS), different loco-regional strategies have been explored in the metastatic setting, namely, for CRC: the survival benefit driven from resection of metastases [4] and the introduction of a multiplicity of locoregional treatment options for distant lesions [5] have progressively encouraged the scientific community to reconsider surgery on a primary tumor in a growing number of patients.

In the metastatic scenario, the management of the primary lesion remains a dilemma in routine clinical practice. The onsite primary gastrointestinal tumor may result in critical complications (such as perforation, digestive tract obstruction or bleeding), impair quality of life (QoL), hinder the administration of chemotherapy and limit the use of antiangiogenic agents [6]. On the other hand, primary tumor resection may be associated with a high risk of perioperative morbidity and mortality and with challenging gastrointestinal symptoms, particularly in rectal cancer patients, as well as GC and PC patients, therefore delaying the initiation of systemic therapy. Of note, from a biological perspective, both preclinical and clinical evidence have so far shown that primary tumor resection may result in a higher rate of systemic cancer spread and growth of pre-existing metastatic sites, due to shedding of circulating tumor cells (CTC), systemic immunosuppression and creation of the so-called “pre-metastatic” niche [7]. It is consequently clear that effective management of the primary tumor should be aimed at reducing the risk of acute complications, allowing the administration of more effective systemic treatments in sequential lines, ultimately prolonging survival and ameliorating QoL. 

Upfront resection is not currently supported by high-level evidence from randomized studies, and major guidelines suggest surgery only for selected advanced CRC or GC patients with impelling symptoms [8,9]. However, if asymptomatic patients (as well as patients responding to chemotherapy) could derive an OS benefit from resection is a matter of debate, and several trials are being planned to confirm findings from retrospective series. In this review, we summarize the current role and anticipate the future potential of surgery on the primary tumor in metastatic CRC, GC and PC.

## 2. Primary Tumor Resection for Metastatic Colorectal Cancer: A Matter of Optimal Timing and Patients’ Selection

The prognosis of CRC patients is mainly determined by the disease stage at the time of diagnosis. Nearly 25% of patients present with distant metastases, and almost 50% will develop metastases after curative surgery. In addition, most metastatic CRC patients are not initially amenable to curative-intent surgery [8]. Therefore, management of the primary tumor in synchronous metastatic disease still represents a key challenge for clinicians involved within the multidisciplinary team.

Palliative resection prior to systemic therapy is indicated in patients with a symptomatic primary tumor. Among asymptomatic patients, resection of the primary tumor is not recommended unless R0 resection of both the primary tumor and metastases is feasible. In such cases, guidelines favor systemic chemotherapy as the preferred initial step of treatment [8,10]. However, prophylactic resection is still under debate, and its effect on OS and QoL is uncertain. Supporters of surgery argue that resection decreases the systemic disease burden and may be associated with the reversal of systemic inflammation [11], possibly making systemic therapy more effective. In addition, upfront surgery might identify occult disease in the peritoneum and may prevent the development of debilitating or life-threating complications, a critical issue with systemic therapy-induced myelosuppression or with the use antiangiogenic drugs (emergency surgery being associated with higher operative morbidity and mortality).

The National Surgical Adjuvant Breast and Bowel Project C-10 (NSABP C-10), a phase II, prospective, multicenter, single-arm study, focused on this topic [12]. The study met the primary endpoint of demonstrating the feasibility of nonoperative management by using fluorouracil, leucovorin, and oxaliplatin (FOLFOX) and bevacizumab in metastatic CRC patients presenting with an asymptomatic intact primary tumor and unresectable metastases. After a median follow-up of 20.7 months, most of the patients (84% of 86 evaluable) did not require surgery or die due to the primary tumor. Only 4 (4.7%) out of 10 interventions performed consisted in emergency surgery, and only 3 patients required permanent stomas. The overall rate of major morbidity related to the intact primary tumor was 16.3% at 24 months. Of note, the 29 centers took more than 3 years to accrue 86 eligible patients, partly reflecting the difficulty in establishing such an aggressive disease as truly asymptomatic. 

Opponents of primary tumor resection rebut that the determining factor for patient survival is the optimal control of the metastatic disease, and therefore, systemic therapy should be the priority in such cases. In addition, patients with metastatic CRC who undergo surgery for primary tumor resection are exposed to a 20–30% risk of postoperative morbidity and a 1–6% risk of perioperative mortality [13]. Moreover, surgery could also alter the host immune response favoring tumor growth in the postoperative period [14]. Some preclinical and clinical data suggest a stimulating effect on the angiogenesis of distant metastases by the removal of the primary tumor, assuming an inhibition of angiogenesis by the primary tumor [15,16,17]. Finally, more active systemic therapies could shrink primary lesions, as well as metastases, reducing the risks of complications related to the presence of an intact primary tumor.

### 2.1. Overview of Available Literature Data

In 2011, Venderbosch and colleagues performed a retrospective analysis of the phase III CAIRO [18] and CAIRO 2 [19] trials [20]. They found that resection of the primary tumor is a prognostic factor for OS (median OS 20.7 vs. 13.4 months, hazard ratio (HR) 0.65, *p* < 0.0001), being an independent determinant of OS in CAIRO 2 and in the subgroup of patients with one metastatic site in CAIRO. A major limitation of this retrospective analysis was the lack of information about the reason for non-resection. A subsequent exploratory subgroup analysis of the CAIRO-3 study (assessing the role of capecitabine and bevacizumab after achieving at least stable disease on six cycles of capecitabine, oxaliplatin and bevacizumab) showed that patients with synchronous disease and a resected primary tumor benefitted most from maintenance treatment, compared to patients with metachronous disease or synchronous disease without a resected primary tumor (median OS 25.0 vs. 24.5 vs. 14.9 months, respectively—*p* < 0.0001) [21].

Previously, some non-randomized, mainly single-center, retrospective studies had been published. The strength of these studies is further limited by the few (if any) data regarding the use of systemic treatments. In 2010, a meta-analysis of 8 retrospective studies showed a survival improvement for patients resected on the primary tumor, with an estimated median standardized difference of 6.0 months (standardized difference 0.55, 95% confidence interval (CI) 0.29–0.82, *p* < 0.001) [22]. However, a Cochrane systematic review did not go in the same direction. It focused on no-surgery versus open or laparoscopic resection of the primary tumor, followed by chemo/radiotherapy, and included seven non-randomized studies, six of which also comprised the meta-analysis of Stillwell and colleagues. According to the Cochrane review, the resection of the primary tumor in asymptomatic patients with unresectable metastatic CRC who received chemo/radiotherapy was not associated with prolonged OS. Median survival ranged from 14 to 23 months in the resection group and from 8 to 22 months in the non-resection chemotherapy group [23].

In 2013, a post-hoc analysis assessed the survival impact of primary tumor resection in patients with unresectable synchronous metastases enrolled in the FFCD 9601 trial [24]. The authors found that the median OS [16.3 (13.7–19.2) vs. 9.5 (7.4–12.5) months, *p* < 0.0001], 2-year OS rate [24% (17–32) vs. 10% (5–21), *p* < 0.0001], median progression-free survival (PFS) [5.1 (4.6–5.6) vs. 2.9 (2.2–4.1) months, *p* = 0.001] and 6-month PFS rate [38% (31–46) vs. 22% (13–34), *p* = 0.001] were significantly higher in the resection group than in the non-resection one. By multivariate analysis, resection of the primary tumor was the strongest baseline parameter independently associated with a longer OS (HR 0.42, 95% CI 0.30–0.60, *p* < 0.0001). These survival differences were maintained when the analyses were restricted to patients with a colonic primary tumor; as expected, a higher proportion of patients with rectal cancer was observed in the non-resection group [25]. A crucial caveat of this exploratory post-hoc analysis is that indications for primary tumor resection before patient enrollment in the FFCD 9601 trial were unknown; this could have led to the inclusion in the resection group of patients both with and without primary related symptoms at diagnosis. The results of the above-mentioned study were confirmed in a pooled analysis of individual data from four randomized trials [26]. All the included studies—that is, the FFCD-9601 trial [24], the FFCD-2000-05 phase III trial [27], the Actions Concertés dans les cancers COloRectaux et Digestifs (ACCORD) 13 trial [28] and the ML-16987 trial [29]—enrolled CRC patients with unresectable metastases; patients who underwent a resection of the primary tumor before starting first-line chemotherapy (478 out of 810) formed the resection group. Resection of the primary tumor was associated with a 6-month longer OS (13.3 vs. 19.2 months, HR 0.53, 95% CI 0.35–0.80, *p* < 0.001). A meta-analysis of 21 retrospective studies found similar results (odds ratio for death 0.28, 95% CI 0.165–0.474, *p* < 0.001, but significant heterogeneity existed) [30], as did a nationwide population-based study (HR of death 0.44, 95% CI 0.35–0.55, *p* < 0.001) [31]. Primary tumor resection was also an independent predictor of PFS, although with a lower effect size (HR 0.82, 95% CI 0.70–0.95, *p* < 0.001). The authors found that the OS benefits were higher in cases of rectal primary or lower baseline carcinoembryonic antigen (CEA) level. As for rectal cancer, this difference could be due to its intrinsic biologic aggressiveness compared to colon cancer, making surgical cytoreduction more effective in this subgroup. However, a selection bias could not be excluded, as proctectomy is more challenging than colectomy and, therefore, is generally performed in more selected patients. As for CEA, a higher level may indicate a higher tumor burden, suggesting that this subgroup of patients might not benefit from surgery [26].

In order to identify reliable selection criteria to guide surgery on the primary tumor, different authors suggested that several parameters could be of help, e.g., secondary curative surgery, well-differentiated primary tumor, liver-limited metastases, good performance status (PS), sequential chemotherapy [22,30,32,33], normal lactate dehydrogenase (LDH) levels and CEA < 70 ng/mL [34] and low preoperative modified Glasgow prognosis score [35]. Other authors built prognostic models based on different clinical and laboratory parameters, such as advanced age (≥65 years); poorly differentiated histology; metastases to liver, lung and bone; peritoneal carcinomatosis; hypoalbuminemia; CEA ≥ 250 ng/mL [36] or advanced age (>70 years); presence of ascites; elevated alkaline phosphatase (ALP); platelet/lymphocyte ratio > 162; and no postoperative therapy [37]. More recently, Li and colleagues found that primary tumor lymph node status was also a strong predictor of cancer-specific survival after palliative resection of metastatic CRC; the advanced nodal stage and limited number of negative lymph nodes were correlated with a higher risk of cancer-related death [38].

As we focus on the role of surgery on the primary tumor in metastatic CTC, we should recognize that significant advances have been achieved in the last decades in terms of systemic therapies and molecular patient selection [39,40,41,42]. Systemic chemotherapy and biologic therapy can control symptoms, prolong OS, improve QoL and, in some cases, convert unresectable disease to a resectable one [8,10]. Possibly as a result of the improvements in medical management of CRC, since 2001, a drop in the resection rate of primary tumors in patients presenting with stage IV CRC has been registered since 2001, passing from 74.5% in 1988 to 57.4% in 2010 (*p* < 0.001). Nonetheless, this less intensive approach to the primary tumor in unresectable stage IV disease has accompanied a doubling in OS median relative survival rates, from 8.6% in 1988 to 17.8% in 2009 (*p* < 0.001) [43]. By the way, using the same datasets (Surveillance, Epidemiology and End Results database between 1998 and 2009), other investigators confirmed that the rate of metastatic CRC patients undergoing primary tumor removal relatively diminished over the past decade. Moreover, they found that the overall and cancer-specific survival improved over time, both for resected and non-resected patients. Finally, this population-based, propensity score-adjusted trend analysis showed that the overall and cancer-specific survival remained significantly higher in patients undergoing palliative primary cancer removal (HR 0.40, 95% CI 0.39–0.42, *p* < 0.001 and HR 0.39, 95% CI 0.38–0.40, *p* < 0.001, respectively), suggesting that the dogma that an asymptomatic primary tumor should not be resected in patients with unresectable CRC metastases could be questioned [44].

In 2018, the largest individual patient data analysis of recent metastatic CRC phase 2 and 3 randomized clinical trials, including targeted therapy comprised in the ARCAD (Aide et Recherche en Cancérologie Digestive) CRC database, was published. The results of this analysis showed that patients with synchronous metastatic CRC and an unresected primary tumor had a worse median OS (16.4 months) compared with resected patients with synchronous (22.2 months, HR 1.60, 95% CI 1.43–1.78) and metachronous distant disease (22.4 months, HR 1.81, 95% CI 1.58–2.07), and this association was proved independent from other variables, such as primary tumor location, liver/lung involvement, number of metastatic sites, body mass index and LDH levels. Moreover, the prognosis of patients with synchronous metastatic CRC without primary tumor resection remained worse, regardless of treatment types (targeted versus non-targeted agents). The authors found that in patients with synchronous metastatic CRC, factors associated with primary tumor resection were female gender, colon tumor, isolated lung or liver involvement, single metastatic site and lower LDH levels (*p* < 0.001). As previously discussed, the main limitation is represented by the fact that the reasons behind the management of the primary tumor were unknown and were not part of the study protocols. Moreover, this analysis excluded patients who did not meet the trial inclusion criteria, for instance, due to complications or death after primary tumor resection, leading to selection bias [45].

In 2018, another two meta-analyses were published about this unresolved issue. Nitsche and colleagues selected 56 retrospective studies for a total of 148,151 patients, finding that primary tumor resection led to an improvement in OS of 7.76 months (95% CI 5.96–9.56 months), but the risk of obstruction and bleeding complications were not reduced (relative risk ratio of 0.50 (0.16 to 1.53) and 1.19 (0.48 to 2.97), respectively). Moreover, as for chemotherapy-related adverse events, an insignificant increase in the group of patients with an intact primary tumor was observed [46]. Ha and collaborators conducted a meta-analysis of 17 non-randomized studies for a total of 18,863 patients treated in the era of modern chemotherapy, focusing also on survival outcomes in different subgroups. Primary tumor resection was associated with a longer OS in patients with unresectable metastatic CRC (HR 0.63; 95% CI, 0.56–0.71; *p* < 0.001). OS improvement was confirmed by the analysis of studies including patients receiving targeted agents and by the analysis of studies in which all patients in the non-resection group received chemotherapy [47]. More recently, another two systematic reviews and meta-analyses were conducted, coming to opposite conclusions [48,49].

The most relevant studies addressing the management of primary tumor and reporting significant results are summarized in Table S1. The retrospective nature of most studies, the risk of selection bias, the lack of data (about treatment allocation; QoL; systemic therapies administered; and baseline prognostic factors related to single-patient, disease status and biologic profile) and the inclusion of patients with a resectable metastatic lesion together with candidates for palliative therapy alone are all factors to be considered when interpreting the results [50,51,52].

Recently, the results of the first randomized, controlled, phase III trial about this topic were published. The iPACS study, designed by the Colorectal Cancer Study Group of the Japan Clinical Oncology Group (JCOG), aimed to confirm the superiority of primary tumor resection plus chemotherapy to chemotherapy alone in asymptomatic stage IV CRC patients with synchronous incurable metastatic disease. The control arm, which did not include primary tumor resection before starting first-line chemotherapy, allowed colorectal surgery in case of occlusion, bleeding, perforation or fistulation. In 9 years, 165 patients were randomized (84 in the standard arm vs. 81 in the experimental arm). The published data refer to the first interim analysis, conducted after 50% of the expected events. The trial was prematurely discontinued due to futility, as no difference in outcome between the 2 arms was observed after 22 months of median follow-up. The median OS in the experimental arm was 25.9 months, compared with 26.7 months in the chemotherapy-alone arm (HR 1.10; 95% CI 0.76–1.59; one-sided *p* = 0.69 by the stratified log-rank test). In addition, 3 deaths were recorded (4%) during the postoperative period in the experimental arm, and a third of patients had surgical morbidity [53]. The results of iPACS are in favor of systemic therapy alone, but again, some missing points should be considered (e.g., molecular biology, post-progression treatments and assessment of QoL), as well as the slow accrual, with only 20% of the initially planned patients enrolled in 7 years. At the 2022 ASCO Annual Meeting the SYNCHRONOUS (ISRCTN30964555, [54]) and CCRe-IV (NCT02015923, [55]), trials were presented. Due to similarity in the eligibility criteria, interventions and endpoints, the data of the 2 studies were pooled (295 patients from SYNCHRONOUS and 98 from CCRe-IV). Both compared primary tumor resection followed by systemic chemotherapy to systemic chemotherapy alone in stage IV colon cancer patients without primary tumor-related symptoms. Notably, the SYNCHRONOUS trial clearly defined tumor-related symptoms and diagnostic findings requiring urgent surgery. No statistically significant differences were observed in OS between resected and non-resected patients (median OS 16.7 months and 18.6 months, respectively; HR 0.95, 95% CI 0.743–1.215, *p* = 0.685) [54]. The full publication of these studies is eagerly awaited.

### 2.2. Ongoing Prospective Trials

Table 1 shows an overview of ongoing randomized controlled trials for primary tumor resection in patients with unresectable stage IV CRC.

Several randomized clinical trials were prematurely closed due to slow accrual, such as the ISAAC trial (NCT01086618), the SUPER (ACTRN12609000680268) trial and a Korean multicenter trial (NCT01978249). A clinical trial carried out at University College London Hospitals has been completed, but results are pending (NCT01086618). Ongoing randomized studies are the Dutch CAIRO4 (NCT01606098, [55,56]), the Chinese NCT02149784 and NCT02291744 trials and the French GRECCAR 8 (NCT02314182, [57]) and CLIMAT-PRODIGE 30 (NCT02363049) trials [58]. All these trials focused on asymptomatic patients. The CAIRO4 and CLIMATE-PRODIGE 30 trials evaluate the role of surgery before the start of a first-line therapy. On the contrary, in GRECCAR-8 and the Chinese trials, surgery is preceded by systemic therapy, and the surgical procedure is considered only in patients without disease progression. GRECCAR 8 enrolls only patients with rectal adenocarcinoma (<15 cm from the anal verge), while the Chinese NCT02291744 trial included solely patients with colon adenocarcinoma. Instead, both colon and rectal cancer (at least 12 cm far away from the anal verge) were included in the Chinese NCT02149784 trial. The CLIMATE-PRODIGE 30 study enrolls only liver-limited metastatic CRC patients. In most of these trials, distant metastases should be judged unresectable by a multidisciplinary team.

Recently, CAIRO4 investigators published preliminary safety results and reported a higher 60-day mortality among patients randomized to primary tumor resection, followed by systemic treatment (11% vs. 3%, *p* = 0.03). In particular, patients randomized to the primary tumor resection group with elevated LDH, neutrophils, aspartate aminotransferase and/or alanine aminotransferase seem to be at increased risk of postoperative mortality [59].

### 2.3. Choosing Wisely: A Tentative Algorithm

As association of resection with OS is not definitively proven, discussion within the multidisciplinary team is always needed in asymptomatic advanced CRC patients before starting first-line therapy. Optimal timing is also a crucial issue in order to maximize the benefit/risk ratio of resection in the single patient. As suggested by available guidelines, in the case of asymptomatic primary CRC, systemic therapy should be offered first, as the risks of complications of the primary tumor may be limited when the optimal combination treatment is administered. In this setting, the aim of systemic treatment would be both to contain disease progression and to select patients for secondary surgery after achieving disease control [8,10]. As the duration of combination therapy in metastatic CRC has been largely investigated in randomized studies, 4 to 6 months of treatment seems the best compromise to achieve maximal shrinkage with an acceptable safety profile [8].

Due to the limitations of the available evidence, defining a validated algorithm for every clinical scenario is not possible, and practical considerations should, therefore, inform treatment decisions within the multidisciplinary team. Tumors arising in the right colon have a worse prognosis, are less frequently the cause of bowel obstruction and generally require a more complex surgical procedure than left-colon cancers. These factors prompt the primary need for effective (and intensive, when feasible) systemic therapies over the risks and possibly more limited benefit of surgery on long-term outcome [60]. Immediate surgery is a choice in the case of symptomatic primary or mildly symptomatic metastatic CRC patients, when the safe administration of effective therapies (e.g., antiangiogenetic drugs) might be impaired. It can also be considered in patients with limited metastatic involvement of distant organs, but who are candidates for less active systemic therapies due to age or comorbidities. Upfront resection may also be an option in patients with potentially resectable metastases, with the aim to offer the most effective systemic therapies available so far and limit the surgical insult of subsequent resection of metastases [39]. An unanswered question is when and how should endoluminal surveillance be performed in asymptomatic metastatic CRC patients with an intact primary tumor: routine endoscopic surveillance may identify imminent problems, providing an opportunity for interval endoluminal intervention or elective resection, and preventing the morbidity risks associated with the need for emergent surgical intervention.

Hopefully, greater importance will be given in the next years to the molecular biology of the tumor, rather than the site per se, by implementing decision algorithms, with the mutational status of the major drivers (e.g., *RAS* and *BRAF*) associated with disease progression in a more accurate prognostic assessment before surgery [8]. A significant example in this context is given by the knowledge of the microsatellite instability (MSI) status. Despite the immaturity of its specific role in the definition of benefit from surgery in the metastatic setting, MSI-high status represents the strongest predictor of immune checkpoint inhibitors (ICI) activity and efficacy. The 9% curative-intent surgery with pembrolizumab in the pivotal Keynote-177 [61] trial and the 67% pathological complete responses of non-metastatic colon cancer patients after neoadjuvant ICI [62] suggest that the use of immunotherapy in this subset of patients could likely lead to a curative perspective selected cases, not excluding a role for surgery.

## 3. Surgery on Primary Tumor in Metastatic Gastroesophageal Cancer: Have We Already Got the Answers We Need?

In the last years, systemic treatment of metastatic GC has witnessed significant advances, thanks to the optimization of chemotherapy treatment, introduction of targeted agents (namely trastuzumab in HER2-positive disease [63]) and availability of salvage treatment for pretreated patients [64,65]. More recently, the advent of immunotherapy is further revolutionizing patient management [66]. Despite these new options, however, the prognosis remains unsatisfactory, with a median OS barely exceeding 12 months in unselected patients starting first-line combination chemotherapy [3]. This is largely due to the early spread of GC to distant organs and the peritoneal cavity. Any attempt to improve the prognosis or QoL by resection of the primary tumor is, therefore, regarded as less effective, compared to adequately addressing distant disease by systemic treatments.

Resection of the primary tumor, with or without metastasectomy, in the context of stage IV disease has been proposed to address two potential indications: (a) symptomatic lesions causing obstruction or bleeding, particularly when nonoperative procedures are difficult or at risk of failure or complications; (b) primary tumor burden reduction (cytoreduction) to prevent the onset of symptoms leading to systemic treatments delay or discontinuation [67]. Such a surgical indication should balance the risks of postoperative complications causing immunosuppression with the benefits of tumor burden reduction. Fortunately, although historically associated with significant morbidity and mortality rates, gastroesophageal cancer surgery shows acceptable postoperative outcomes nowadays, at least in high-volume centers [68], with Asian authors reporting lower postoperative mortality compared to Western authors [69]. Moreover, together with advances in surgical techniques, improvements in nutritional support and anesthetic procedures contributed to lowering the risk and enhancing the recovery of GC patients after surgery [70]. Nonetheless, the American College of Surgeons has recently addressed this specific question, showing that GC surgery in the setting of metastatic patients (377 cases) was more commonly hampered by major morbidity (HR 1.49; 1.16–1.90), namely, respiratory events (HR 1.58; 1.07–2.33), 30-day mortality (HR 2.19; 1.38–3.48) and prolonged hospital stay (HR 1.65; 0.31–2.07) [71].

### 3.1. Overview of Available Literature Data

The most relevant data on palliative resection (with or without systemic treatment) over systemic therapy alone are outlined in Table 2. Randomized and retrospective evidence on the Western population is hereafter discussed. The flourishing number of retrospective publications in the last five years witnesses the attempt to refine the therapeutic strategy in the metastatic setting also for this prognostically disfavored population.

The most relevant data are constituted by different queries of the US National Cancer Data Base (NCDB). In 2021, metastatic GC patients who received either no treatment, palliative chemotherapy alone or palliative gastrectomy (PG) +/− chemotherapy from 2010 to 2015 were analyzed by Kamaraja S.K. et al. [77] against the endpoint of OS. Six percent (1101) of the included patients received PG, being younger, less comorbid, more frequently node-positive and followed in academic institutions. Only 5% of them received post-PG chemotherapy. PG granted a significantly longer OS with respect to chemotherapy alone and no treatment (12.8 vs. 9.5 vs. 1.8 months), which was confirmed for all node subgroups and metastatic sites (liver, bone and peritoneum, >1 sites) but the lung and brain. It is relevant to note that the frequency of PG progressively decreased from 2010–2011 to 2014–2016.

Another two significant population-based retrospective cohorts have been described from NCDB. Warschkow and colleagues compared metastatic GC patients who underwent PG and chemotherapy to those who received only palliative chemotherapy from 2006 to 2012: the former group experienced a significantly longer OS (13.9 vs. 7.9 months; HR 0.60, 95% CI, 0.56–0.64; *p* < 0.001), a higher 2-year OS rate (34.2% vs. 12.6%; HR for resection 0.52; 95% CI 0.47–0.57; *p* < 0.001) and an improvement of all tested QoL endpoints [75]. Picado and collaborators interviewed the same dataset to select patients with liver-metastatic GC, stratified according to treatment received: palliative chemotherapy alone versus gastrectomy with perioperative chemotherapy, with or without hepatic metastasectomy. Of 3175 identified cases, only 196 (6%) belonged to the second group and had a significantly longer median OS (16 versus 9.7 months, *p* < 0.001) and higher survival rate at 1, 3 and 5 years (65%, 27% and 12% in gastrectomy with perioperative chemotherapy group vs. 41%, 9% and 5% in the palliative chemotherapy group, respectively; *p* < 0.001). Among the factors identified at univariate analysis, those associated with longer survival in the Cox model were African-American ethnicity and gastrectomy (HR 0.53, 95% CI 0.44 to 0.63, *p* < 0.001). Of note, at the subgroup analysis of gastrectomy with perioperative chemotherapy, patients treated with additional hepatic metastasectomy showed a non-statistically significant trend toward longer OS if compared to those who underwent gastrectomy alone (24.3 versus 15.3 months, *p* = 0.075) [76].

A multicenter Western cohort study including 3202 patients showed that several factors were independently associated with worse prognosis after PG, such as American Society of Anesthesiologists’ high-risk score, poor PS, solid-organ metastases, peritoneal carcinomatosis (either localized or diffuse) and signet-ring cell histology [80]. Nonetheless, patients without negative prognostic factors were found to reach interesting long-term OS results, mainly when compared to non-resected patients treated with systemic therapy alone.

In a large cohort of asymptomatic stage IV gastroesophageal cancer patients receiving at least two lines of therapy, Italian investigators reported a longer OS among patients treated with PG in addition to chemotherapy (median: 18.7 vs. 13.5 months, respectively; *p* < 0.001) [74]. However, at the subgroup analysis, none of the investigated parameters, including PS, number and sites of metastases, primary tumor location, Lauren’s histology and clinico-pathologic subtype [81], revealed a significant interaction with benefit from surgery. This suggests that available clinical and molecular factors might have limited the utility of the selection of GC patients for an onco-surgical approach.

With the aim to tentatively define the metastatic GC subset of Western patients more likely to benefit from PG, the presence of ≥2 metastatic sites and a preoperative score >28 (built from clinical and biochemistry characteristics) were identified as negative predictors for patients belonging to the American SEER database [82,83].

Finally, two recently published meta-analyses, both updated to 2013, tried to summarize the evidence in support of surgery in the setting of metastatic GC. Sun and colleagues identified 14 studies, with 3003 eligible patients included in the analysis. The authors reported a weighted average of median OS of 14.96 months with gastrectomy and 7.07 months without surgery (HR 0.56; *p* < 0.002) [72]. The OS advantage with surgery was retained in all the explored subgroups, i.e., among patients with hepatic metastases (HR 0.41, 95% CI 0.30–0.55, *p* < 0.00001) and in cases with distant nodal spread (HR 0.36, 95% CI 0.23–0.59, *p* < 0.00001) and peritoneal involvement (HR 0.76, 95% CI 0.63–0.92, *p* = 0.005). Of note, the association of surgery and palliative chemotherapy resulted in a superior OS compared to surgery alone (HR 0.63, 95% CI 0.47–0.84, *p* = 0.002). In addition, a subsequent work of over 19 non-randomized studies including 2911 patients confirmed the putative role of surgery over observation (OR 4.9, 95% CI 3.2–7.5, *p* < 0.0001) or non-resectional approaches (OR 2.6, 95% CI 1.7–4.3, *p* < 0.0001) [73]. Notably, most trials reported a longer OS combining PG and chemotherapy over single-treatment modalities alone.

To summarize, all these studies suggest a mild benefit of primary tumor resection in a subset of patients with metastatic GC. However, there are some criticisms that do not allow adopting such indications in daily clinical practice. First, most of the cited studies do not report whether primary tumor resection was due to the presence of symptoms or not. Moreover, the observed survival advantage could be due to selection bias; indeed, in many retrospective series, patients with a limited metastatic burden and better PS were selected for resection. Last, in some of the above-mentioned studies, surgery included not only gastrectomy, but also the resection of metastatic sites. As such, the survival advantage associated with surgery might not reflect the impact of resecting the primary tumor only.

We can then conclude that if a symptomatic primary tumor is detected in a fit patient with limited disease burden, primary tumor resection is to be considered since it could lead to better survival and increase the chances of receiving effective systemic treatment. On the other hand, the issue of whether to resect an asymptomatic primary tumor in metastatic GC cannot be solved on the bases of retrospective data alone. Consequently, the randomized phase III REGATTA trial was designed in order to answer this clinical question. This study evaluated the role of surgery among 175 asymptomatic metastatic GC patients and a single incurable factor defined as follows: 2–4 hepatic metastases with a diameter between 1 and 5 cm; peritoneal metastases in the diaphragm or peritoneum caudal to the transverse colon not associated with refractory ascites or intestinal obstruction; para-aortic lymph node metastases above the celiac axis and/or below the inferior mesenteric artery [78]. REGATTA was conducted among 44 Asian centers and patients randomly assigned to gastrectomy followed by chemotherapy or chemotherapy alone. Systemic therapy consisted of the standard fluoropyrimidine (with S-1) plus cisplatin combination. Unfortunately, the first interim analysis prompted the closure of the trial due to futility. The median OS was 14.3 months in the experimental arm, compared with 16.6 months in the chemotherapy alone arm (HR 1.09, 95% CI 0.78–1.52; one-sided *p* = 0.70). Notably, at subgroup analyses, a significant interaction was reported between treatment effect and clinical nodal stage or tumor location: gastrectomy seemed to be associated with a detrimental effect in clinical N0-N1 disease (HR 1.79, 95% CI 1.14–2.83; *p* = 0.011) and in cases with upper-third tumors (HR 2.23, 95% CI 1.14–4.37; *p* = 0.017), whereas an insignificant trend in favor of surgery plus chemotherapy was observed in the clinical N2-N3 stage and lower-third lesions. However, these findings should be interpreted with caution, as the number of patients in different subgroups is limited and the compliance with chemotherapy was suboptimal in patients with upper tumors submitted to surgery (indirectly reflecting the heavier surgical impact of resection for upper GC or junctional tumors). A reasonable question on the suitability of the aforementioned results for Western patients may be raised due to the well-known biological differences among GC affecting Eastern and Western people [84,85]. Another major criticism of the study relies on its design, assigning patients to immediate resection followed by chemotherapy. Lessons learned in decades of negative adjuvant chemotherapy trials in localized disease confirmed that the tolerability of chemotherapy is impaired after gastrectomy, and REGATTA found that delivery of systemic treatment was suboptimal, at least among patients with resected upper GC. It is, therefore, intuitive that administering systemic therapy first: (i) increases the percentage of patients treated with adequate dose intensity, (ii) reveals tumor biology by the identification of responsive patients and (iii) ultimately helps in the selection of surgical candidates by the exclusion of those cases with rapidly progressing disease, who are best treated with palliative measures.

On the bases of REGATTA, the first choice in asymptomatic metastatic GC should be chemotherapy. This negative answer led to a subsequent clinically relevant question, i.e., whether surgery should be considered in patients who obtain an objective response during systemic treatment, and consequently, whether surgery should be limited to primary tumor resection or extended to metastasectomy. In this regard, the phase II FLOT3 trial from the German AIO group evaluated the outcome in patients with limited metastatic disease (i.e., abdominal, retroperitoneal lymph node metastases only, or one incurable organ site with or without retroperitoneal lymph node metastases, and no clinically visible or symptomatic carcinomatosis of peritoneum or pleura and no diffuse peritoneal carcinomatosis on diagnostic laparoscopy, and fewer than five liver metastases, if the single organ site is the liver) receiving intensive chemotherapy with the triplet FLOT schedule (infusional 5-fluorouracil, oxaliplatin and docetaxel) [79]. This trial enrolled 252 patients with resectable or metastatic gastric or gastroesophageal carcinoma, stratified in 3 groups according to the extent of disease (i.e., resectable, limited metastatic or extensive metastatic). Of 60 patients with limited metastatic disease amenable to resection of the primary tumor and metastases in the case of response, 36 underwent surgery. The median OS was 31.3 months for resected patients, apparently longer than that reported with FLOT alone (15.9 months). Notably, the trial was not designed to compare chemotherapy followed by surgery over chemotherapy alone, but results are encouraging and seemed to suggest that the best choice in the setting of limited, resectable metastatic GC is the radical-intent surgery on the primary tumor and metastatic site, as well, providing a rationale for further investigations.

### 3.2. Ongoing Prospective Trials

On the bases of FLOT3, the RENAISSANCE (AIO-FLOT5) trial was planned. This is a prospective, multicenter, randomized, investigator-initiated phase III trial aiming to evaluate the effects of perioperative chemotherapy with FLOT in chemo-naïve patients with limited metastatic (i.e., same as AIO-FLOT3, with additional specifications enlisted in the protocol) adenocarcinoma of the stomach or esophagogastric junction (without prior tumor resection), in combination with curative gastrectomy/esophagectomy, plus resection of metastatic lesions or the local ablation procedure. Patients without disease progression after 4 cycles of chemotherapy (FLOT alone or with trastuzumab if HER2-positive) are randomized 1:1 to receive additional chemotherapy cycles or surgical resection of the primary tumor and metastases, followed by subsequent chemotherapy. The primary endpoint is OS [86].

Further evidence on a similar subset of metastatic GC patients is awaited from the ongoing French SURGIGAST trial (NCT03042169). This randomized phase III trial is aimed at comparing upfront resection of the primary tumor and metastasectomy or locoregional treatment of metastatic sites, followed by chemotherapy, versus a standard chemotherapy-alone arm against the primary endpoint of OS and the secondary outcomes of toxicities and complications. Here, oligometastatic patients are defined as having a locally resectable primary tumor and retro-peritoneal lymph node metastases and/or another metastatic lesion on only one organ (solid organ, lymph node or limited localized peritoneal carcinomatosis with peritoneal cancer index < 7). The primary completion date is estimated as February 2023.

Despite not being prespecified in the trials’ design, the results of these studies with respect to the MSI population are highly awaited to define the role of surgery in the GC metastatic setting. Indeed, this rare population is known to derive greater benefit from surgery, without [87] or with [88] neoadjuvant immunotherapy, than from chemotherapy in the early stages, and from the combination of chemotherapy and immunotherapy over chemotherapy alone in the metastatic setting [89], thus lacking a specific knowledge for surgery in the advanced stage.

### 3.3. Putting Data in Context

To sum up the so-far available evidence, we believe that a modern approach to metastatic GC should take into consideration also the surgical strategy, especially when aimed at R0 resection (thus, comprising surgery both on the primary and metastatic sites). However, the lack of a definitive proof of survival benefit from PG, with or without metastasectomy, should be recognized. In light of the advances in the medical management of advanced disease, systemic treatments are the key in all asymptomatic patients, and the risks and benefits of surgery should be discussed for selected cases only, within a well-trained multidisciplinary team. Moreover, non-surgical techniques could represent effective alternatives to resection for prompt symptom palliation in order to minimize risks and speed up treatment initiation.

As proposed elsewhere [90], an oncosurgical approach should be pursued in biological and technical favorable cases, i.e., metastatic GC with para-aortic metastatic lymph-nodes and/or a single hepatic lesion <5 cm of diameter and/or cytologically positive peritoneal disease without macroscopic peritoneal lesions. For GC patients with a higher disease burden, systemic therapies remain the mainstay of treatment in all cases. In the near future, improvements in treatment activity with chemo-immune combinations could open the way to reconsider surgery in a larger percentage of patients and candidates for conversion surgery, and trials are ongoing to address this issue (such as the Eastern NCT04267549, NCT04694183, NCT05177068 and NCT04886193).

## 4. Surgery on Primary Tumor in Metastatic Pancreatic Cancer: Onco-Surgical Fancy or Reasonable Multidisciplinary Approach in Selected Patients?

Most pancreatic cancer (PC) patients present with metastatic disease at the time of diagnosis [91]. Despite recent improvements linked to the introduction of more effective chemotherapy regimens in the metastatic setting, the prognosis remains disappointing, reaching 11.1 months median OS with FOLFIRINOX [92] and 8.5 months with gemcitabine plus nab-paclitaxel [93] in phase III trials. PC incidence is increasing, and according to recently reported epidemiologic data, it will become the second leading cause of cancer-related death in Western countries by 2030 [94]. Preclinical studies on mice models indicate that PC cells may enter the bloodstream and invade other organs very early, even before a frank malignancy can be detected by histological analysis of the pancreatic glands [95]. Therefore, the role of local therapies in this disease should always be carefully evaluated, and a call for new clinical approaches has been advocated [96]. However, the improvements in the treatment of metastatic disease resulted in higher chances of objective response and prolonged OS in an increasing (despite limited) percentage of patients [97]. Moreover, the results of randomized studies evaluating adjuvant treatment after surgery show that chemotherapy may cure a significant group of patients with micrometastatic disease and that a more active chemotherapy may even enlarge this benefit [98,99,100]. Furthermore, even if PC frequently spreads to the liver, recent data suggest heterogeneity in its clinical and biological behavior among patients, with different patterns of metastatic progression and a better prognosis observed in patients with lung metastases [101]. Similar heterogeneity has been demonstrated in the genetic landscape of PC, identifying different subgroups that may also require different treatment strategies [102].

Primary PC may cause relevant symptoms, such as pain, jaundice, bleeding and duodenal obstruction, requiring palliative local treatments anyway. The mortality of surgical procedures for PC removal in qualified centers is not very different nowadays from that reported with palliative interventions to treat these symptoms [103]. Therefore, even in this aggressive disease, the rationale for surgical removal of the primary tumor in selected metastatic patients may be sustained.

### 4.1. Overview of Available Literature Data

No randomized trial on this topic has been published so far. The multicenter phase 3 randomized Chinese “Simultaneous Resection of Pancreatic Cancer and Liver Oligometastasis After Induction Chemotherapy—CSPAC-1” trial, which aims at enrolling 300 participants, is ongoing since 2018. With the limit of the geographic selection, this study will tackle the question of whether synchronous resection of primary pancreatic cancer and liver oligometastasis (defined as ≤3 lesions) at maximum radiological response on first line FOLFIRINOX or gemcitabine + nab-paclitaxel/S-1 will result in a prolonged OS over chemotherapy. Recruitment completion is awaited by early 2025 (NCT03398291).

The largest observational evidence on this topic comes from a SEER-based analysis published in 2021 [104]. The 15,836 stage IV patients diagnosed between 1976 and 2016 were compared as follows: chemotherapy with or without primary tumor resection (9515), chemoradiation with or without primary tumor resection (699) and no treatment versus primary tumor resection only (5403). With the limitation of the lack of detailed information regarding systemic treatments, improved OS and cancer-specific survival (CSS) were described for chemotherapy plus primary tumor resection versus chemotherapy (median OS: 13 vs. 9 months, *p* = 0.024; median OS CSS: 14 vs. 10 months, *p* = 0.035) and chemoradiotherapy plus primary tumor resection versus chemoradiotherapy (median OS: 14 vs. 7 months, *p* = 0.044; median OS CSS: 14 vs. 7 months, *p* = 0.066), and they were confirmed at multivariate analysis. Of note, primary tumor resection significantly improved the OS and CSS among patients with ≤1 metastatic organ, thus suggesting a specific selection criterion for this approach.

In addition to this evidence, different retrospective series have been published across the years [105,106], reporting disappointing results in terms of median OS, usually lower than or approximately equal to 1 year. More recently, case reports on metastatic PC patients treated with intensified chemotherapy, such as FOLFIRINOX, with apparently complete response on metastases, followed by surgical resection of the primary tumor, have been published [107,108]. In these reports, an interesting OS of more than 2 years has been observed, but the number of evaluated patients is too limited to derive any conclusion. A larger study published by the University of Pittsburgh Medical Center and the Johns Hopkins Hospital reported on 1147 metastatic PC patients, of which 23 (2%) underwent surgical resection of the primary tumor with (11 cases) or without (12 cases) metastasectomy after favorable response to chemotherapy [109]. Most patients received FOLFIRINOX before surgery. The median OS from surgery was 18.2 months (95% CI 11.8–35.5), and the median OS from initial diagnosis was 34.1 months (95% CI 22.5–46.2). Interestingly, 6 out of 23 patients had no recurrence after a median follow up of about 2 years, while the other 7 patients experienced early recurrence within 6 months from surgery.

In this context, the literature devoted to the subgroup of liver-only metastatic PC patients deserves a specific mention, as the liver is the most frequent metastatic localization of PC. The widest evidence comes from the 891 patients who underwent primary surgery and from the 137 patients who underwent pancreatectomy plus liver metastasectomy between 2010 and 2015, according to the National Cancer Database [110]. If compared to non-surgical patients, the former were younger, with lower Ca 19.9 levels, and had a higher median OS (10.74 months vs. 3.4 months *p* < 0.001), confirmed after controlling for patient and disease-related factors (HR: 0.5, 95% CI: 0.4–0.6; *p* < 0.001). Of the latter group, patients receiving chemotherapy, pancreatectomy and hepatectomy had a longer median OS (15.6 months vs. 8.1 months) compared to those who received chemotherapy alone (*p* < 0.001), after propensity score matching.

Smaller case series are quite numerous and report consistent results. Retrospective data from two Italian institutions described a median OS of 39 months among 11 metastatic PC resected on the primary tumor, with (4 cases) or without (7 cases) metastasectomy, after response to different chemotherapy regimens [111]. Of note in this report only, one patient had no recurrence at the end of observation (24 months after surgery). The authors highlighted a potential benefit in OS for resected patients, when compared with a parallel group of patients with similar characteristics and good response to chemotherapy, but without primary tumor resection. At multivariate analysis, several factors were identified as independent predictors of improved OS (chemotherapy with multiple agents and surgical resection) or poorer OS (>5 liver metastases at diagnosis and Ca 19.9 reduction <50% of baseline value). Tachezy and colleagues added other data to the debate, reporting their multicenter retrospective analysis including 69 patients who underwent PC resection with simultaneous surgery on liver metastases [112]. The authors observed a better OS with surgery, in comparison to patients submitted to exploratory procedures only. At subgroup analysis, a survival gain was reported only for PC located in the head of the pancreas (median OS: 13.6 vs. 7 months; *p* < 0.001), whereas patients with body- or tail-located PC showed no advantage (median OS: 14 vs. 15 months; *p* = 0.312).

Of interest are the results presented by Frigerio and collaborators. The role of surgery on PC was retrospectively studied in the selected group of patients with liver only metastases, who achieved disease downstaging after primary chemotherapy. All patients with complete response of liver metastases and significant reduction of Ca 19.9 underwent surgery on the primary tumor site. Disease-free survival (DFS) and OS after diagnosis were as high as 27 and 56 months respectively, while DFS after surgery was 13 months. Despite lacking a true control group, the authors highlighted the potential role of surgery in the specific (despite very limited) subset of metastatic PC patients fully respondent to systemic chemotherapy [113].

### 4.2. Finding the One in a Billion: Is It Really Possible to Select?

Considering the safety of pancreatic resection in adequate centers and the survival data from the outlined series, the main question now remains how to identify metastatic PC patients who may achieve longer OS after primary tumor resection. Certainly, these patients need to be highly selected (2% of all metastatic patients in the larger available series [109]), but no reliable prognostic features are available. Moreover, the absence of a prospectively collected and randomly assigned control group in the reported studies does not allow concluding that the longer OS achieved is due to surgery and not to the biological selection of patients with a better prognosis [114]. While waiting for prospective controlled studies, which will be very difficult to conduct in any case, surgery on a primary tumor should not be considered a standard approach in metastatic PC patients.

Future trials should focus on very special cases with good PS and complete (or nearly complete) response to chemotherapy on metastatic lesions (particularly in cases with lung or lymph nodes metastases), after an adequate period (i.e., at least 6 months) of chemotherapy. Indeed, the cited series achieved the best results when strict criteria were applied, aiming at selecting those patients with a chemo-sensitive disease and more limited distant spreading. All the aforementioned factors have been taken into account already by Herman and colleagues when suggesting their therapeutic algorithm for oligometastatic PC. In their opinion, surgery on the primary tumor is to be considered for patients with low-volume metastatic disease, good PS and a favorable response to intensive systemic therapy [115].

## 5. Conclusions

We have witnessed significant advances in the management of metastatic gastrointestinal malignancies in the last decade. OS improvements have been particularly evident for CRC, and more recently GC. Indeed, the evolution of systemic therapy in gastrointestinal tumors raises crucial questions about the optimal management of a primary tumor. Thanks to the lower surgical insult and more favorable outcome, surgery on a primary lesion should be considered in selected patients with metastatic CRC, with a special focus on candidates for a potentially curative onco-surgical approach on both colorectal and metastatic tumors. However, even for never-resectable distant disease, surgery on the primary tumor may prevent acute complications or adverse events from antiangiogenics and ultimately improve the administration of systemic therapies in the continuum of care and medical treatments.

GC and, above all, PC offer greater challenges to surgeons, considering the higher risks of perioperative mortality and postoperative morbidity compared to colorectal resection. Surgery on the primary tumor should be discouraged as routine practice in metastatic PC; this approach should be strictly reserved for highly motivated patients with limited disease burden, who achieve prolonged distant disease control with chemotherapy in the context of clinical trials. For advanced GC, considering the negative results of REGATTA, gastrectomy is not recommended as the first approach to asymptomatic stage IV disease. Whether surgery can definitively confer an OS advantage (especially in those cases with limited metastatic involvement of distant organs or the peritoneum and responding to first-line therapy) is under investigation; several prognostic parameters have been suggested and, while not representing parts of a stiff algorithm, could guide the decision for the single patient within the multidisciplinary team.

In the last years, the biological basis of different gastrointestinal diseases has been investigated and molecular subgroups of CRC, GC and PC have been identified [102,116,117]. Molecular selection is now entering the routine clinical management of gastrointestinal cancer patients, but it is not considered by surgeons in the decision process, and it was not explored in the available literature discussed in this review. In the future, it is desirable that the biologic events behind a more aggressive or more favorable disease course will soon also guide the multidisciplinary discussion about the management of primary tumors in order to ultimately personalize the therapeutic proposal in different gastrointestinal cancers.

## Figures and Tables

**Table 1:** Ongoing randomized trials about primary tumor resection in metastatic CRC.

Study (ClinicalTrials.gov ID)	Phase	Main Inclusion Criteria	Estimated Enrollment	Study Start Date-Status	Primary Endpoint	Secondary Endpoint	Active Comparator	Experimental Arm
**China, Guangdong (NCT02149784)**	3	Colon cancer or rectal cancer with at least 12 cm far away from anal verge with unresectable metastases. No evidence of obstruction, bleeding or perforation. Pts must respond to 1st line CT.	480	September 2015 Recruiting	3-years OS	Number of pts with AEs both in surgery group and CT group.	Unresectable mCRC pts responders to CT will continue with CT	Unresectable mCRC pts responders to CT will receive surgical resection of PT
**China, Shanghai** **(NCT02291744)**	2	Colon cancer adenocarcinoma. Primary and metastatic tumors exist at the same time, and distant metastases are not resectable. No need of surgery for perforation, bleeding or obstruction. No uncontrollable large pleural or peritoneal effusion. No brain metastases.	130	October 2014 Recruiting	TFS	None.	8 cycles of XELOX	8 cycles of XELOX plus surgery
**CAIRO4** **(NCT01606098)** Denmark and Netherlands, multicenter	3	Resectable PT in situ (CRC) with unresectable distant metastases. No indication for neo-adjuvant (chemo)radiation. No signs or symptoms PT-related that require immediate intervention (i.e., surgery, stenting, systemic therapy or radiotherapy). No condition preventing the safety or feasibility of resection of the PT (i.e., massive ascites or extensive peritoneal disease).	360	July 2012 Active, no longer recruiting	OS	PFS. RR. G3-4 CT-related toxicity. Surgery-related morbidity and mortality. QoL. Interval between randomization and initiation of CT. Cost-benefit analyses. Pts requiring resection of PT in the non-resection arm.	1st line FP-based CT with bevacizumab	PT resection followed by 1st line FP-based CT with bevacizumab
**GRECCAR 8** **(NCT02314182)** France, multicenter	3	Rectal adenocarcinoma (<15 cm from the anal verge) with few or no symptoms and unresectable synchronous metastasis not amenable to curative treatment. No known unresectable PT (with clear margin > 1 mm) on imaging. No PD under CT (for at least 4 cycles). Assessment of KRAS status. No peritoneal carcinomatosis.	290	November 2014 Completed (trial end date: 27 February 2018): no longer recruiting	OS	PFS. QoL. Toxicity of CT. RR. Time to PD. Postoperative morbidity.	Continued systemic CT ± target therapy	Immuno-nutrition, PT resection + systemic CT ± target therapy
**CLIMAT-PRODIGE 30** **(NCT02363049)** France	3	Colon adenocarcinoma (≥15 cm from the anal verge) Uncomplicated PT. No known unresectable PT on imaging. Unresectable synchronous liver metastases. No extra-hepatic metastatic disease.	278	July 2014 Recruiting	OS	QoL Postoperative complications. PFS. TTP. Rate of secondary curative resection (R0).	CT ± targeted therapy alone	Surgery followed by CT ± targeted therapy

AEs: adverse events; CT: chemotherapy; FP: fluoropyrimidine; mCRC: metastatic colorectal cancer; OS: overall survival; PD: progression of disease; PFS: progression-free survival; PT: primary tumor; pts: patients; QoL: quality of life; R0: complete tumoral resection; RR: response rate; TFS: time of failure of strategy (the second progression time after induction therapy, time to the use of second line strategy (if no reapplication of induction therapy) or time to no further treatment); TTP: time to metastatic progression; XELOX: capecitabine/oxaliplatin.

**Table 2:** Most relevant studies about primary tumor resection in metastatic GC.

Study (Year of Publication) [Ref.]	Design	No.	OS (Months or Survival Rate, %)	HR/OR (95% CI)	Subgroup Analysis: HR (95% CI) or *p*-Value for OS
Sun J. et al. (2013) [72]	Meta-analysis (published data)	3003	Weighted average of median OS: Gastrectomy: 14.96 Control: 7.07	0.56 (0.39–0.80)	Peritoneum: 0.76 (0.63–0.92) Liver: 0.41 (0.30–0.55) Lymph node: 0.36 (0.23–0.59)
Lasithiotakis K. et al. (2014) [73]	Meta-analysis (published data)	2911	1-year OS: Gastrectomy: 50% (weighted mean) Non-resectional surgery: 10% Control: 39%	2.6 (1.7–4.2) 4.9 (3.2–7.5)	Not reported
Yazici O. et al. (2016)	Retrospective	488	Median OS: Gastrectomy: 14 Control: 9	0.52 (0.38–0.71)	Peritoneum: *p* < 0.001 Visceral metastases: *p* < 0.001
Fornaro L. et al. (2017) [74]	Retrospective	513	Median OS: Gastrectomy: 18.7 Control: 13.5	0.620 (0.487–0.790)	Peritoneum: 0.52 (0.35–0.77) Liver: 0.71 (0.48–1.06)
Hsu J.T. et al. (2017)	Retrospective	333	Median OS: Gastrectomy + metastasectomy: 7.7 Non-resective procedures: 4.9	*p* < 0.001	Age (> vs. ≤58 years): 1.47 (1.01–2.13) Albumin (> vs. ≤3 g/dL): 1.93 (1.24–3.00) N1/N0: 0.83 (0.33–2.10) Adjuvant CT (no vs. yes): 1.68 (1.19–2.38)
Warschkow R. et al. (2018) [75]	Retrospective population-based cohort	7026	Median OS: Primary tumor resection + CT: 13.9 CT: 79	0.60 (0.56–0.64)	Lymph node: 0.52 (0.41–0.66) Peritoneum: 0.66 (0.53–0.83)
Picado O. et al. (2018) [76]	Retrospective population-based cohort	3175	Median OS: Gastrectomy with perioperative CT: 16 CT: 9.7	0.53 (0.44–0.63)	African American: 0.81 (0.71–0.91) Non-academic program: 1.23 (1.13–1.33) Overlapping lesion: 1.23 (1.11–1.37) Moderately differentiated: 1.18 (0.90–1.56)
Kamarajah S.K. et al. (2021) [77]	Retrospective population-based cohort	19,411	Median OS: No treatment: 1.8 CT: 9.5 Gastrectomy: 12.8	0.76 (0.71–0.81) vs. CT	N0: 0.66 (0.56–0.77) N1: 0.65 (0.56–0.76) N2: 0.80 (0.64–1.00 N3: 0.76 (0.60–0.97) Liver: 0.82 (0.72–0.93) Peritoneum: 0.59 (0.37–0.95) Lung: 1.07 (0.78–1.69) Bone: 0.56 (0.36–0.89)
Park J.Y. et al. (2021)	Retrospective	148	Median OS: Palliative gastrectomy: 28.4 Non-resection: 7.7	*p* < 0.001	Not reported
Fujitani K. et al. (2016) [78]	Randomized phase III	175	Median OS: Gastrectomy + CT: 14.3 CT: 16.6	1.09 (0·78–1·52)	N0-1: 1.79 (1.14–2.83) Upper-third tumors: 2.23 (1.14–4.37)
Al-Batran S.E. et al. (2017) [79]	Phase II (subgroup analysis)	60	Median OS: Gastrectomy + metastasectomy: 31.3 months Control: 15.9 months	Not reported	Not reported

No.: number of patients; HR/OR (95% CI): hazard ratio/odds ratio (95% confidence interval); OS: overall survival; CT: chemotherapy; N: nodal status.

## Supplementary Materials

The following supporting information can be downloaded at: https://www.mdpi.com/article/10.3390/cancers15030900/s1, Table S1: Most relevant studies addressing the management of primary tumor in patients with metastatic colorectal cancer. References [13,20,25,26,31,32,33,44,118,119,120,121,122,123,124,125,126,127,128,129,130,131,132,133,134,135,136,137,138,139,140,141,142,143] are cited in the Supplementary Materials.
