# Supplementary material for: Primary Tumor Resection for Metastatic Colorectal, Gastric and Pancreatic Cancer Patients: In Search of Scientific Evidence to Inform Clinical Practice
Source: Cancers (Basel). 2023 Jan 31;15(3):900. doi: 10.3390/cancers15030900 (PMC9913845; doi:10.3390/cancers15030900)
Supplement: Supplementary file 1 [file cancers-15-00900-s001.zip › cancers-2149272-supplementary.pdf]

Table S1. Most relevant studies addressing the management of primary tumor in patients with metastatic colorectal cancer

Study	Study type	Enrollment interval	Patients		Rectal cancer (% of all patients)	Median overall survival (months)			Chemotherapy regimens
			Resection group (N)	Non-resection group (N)		Resection group	Non-resection group	P value	
Xu et al.	Retrospective, large database	1988-2010	27931	16583	34	16	7	HR 0.45 (0.44-0.46)	CT regimen not specified
Konyalian et al.	Retrospective, single center	1991-2002	62	47	26	12.5	4.6	<0.001	NR
Ahmed et al.	Retrospective cohort study	1992-2005	944	434	30	Resection of PT + CT: 18.3	Only CT: 8.4	<0.0001	5-FU–based chemotherapy
Ahmed et al.	Retrospective	1992-2005	521	313	33	19.7<br>29.4	8.4<br>16.0	<0.0001<br><0.001	5-FU-LV<br>Oxaliplatin- or irinotecan-based
Beham et al.	Retrospective, single center	1993-2003	46	21	43	18.1	7.8	<0.001	CT regimen not specified
Duraker et al.	Prospective cohort study	1993-2004	110	54	46	11.0	5.5	<0.001	5-FU-based
Costi et al.	Retrospective, single center	1994-2003	83	47	21	9	4	<0.001	No CT.
Stelzner et al.	Retrospective, single center	1995-2001	82	25	39	11.7	5.2	0.0002 (univariate)	5-FU-based
Galizia et al.	Retrospective, single center	1995-2005	42	23	18	15.2	12.3	0.03	5-FU-based/Oxaliplatin/Irinotecan
Ruo et al.	Retrospective, single center	1996-1999	127	103	32	16.0	9.0	<0.001	CT regimen not specified<br>Radiation
Mik et al.	Retrospective, single center	1996-2000	52	82	48	21	14	0.004	5-FU-LV
Tsang et al.	Retrospective, large database	1996-2007	8599	3117	20	21	10	<0.0001	CT regimen not specified
Ferrand et al.	Post-hoc analysis of a multicenter RT	1997-2001	156	60	20	16.3	9.5	<0.0001	5-FU-LV ± Raltitrexed
Ishihara et al.	Retrospective, multicenter	1997-2007	1782	200	23	17.2 (CSS)	6.4 (CSS)	<0.01	CT regimen not specified
Faron et al.	Pooled analysis of individual data from four RT	1997-2008	478	332	18	19.2	13.3	<0.001	5-FU-LV/Raltitrexed/FOLFOX/ FOLFIRI ± bevacizumab/ XELIRI + bevacizumab/
Kaufman et al.	Retrospective, single center	1998-2003	115	69	28	22.0 (surgery)<br>30.0 (surgery + CT)	3.0 (no CT)<br>15.0 (CT)	<0.0001<br><0.00041	CT regimen not specified
Aslam et al.	Retrospective, single center	1998-2007	366	281	35	14.5	5.8	<0.001	CT regimen not specified
Karoui et al.	Retrospective, multicenter	1998-2007	85	123	0	30.7	21.9	0.031	Oxaliplatin-or irinotecan-containing ± anti-VEGFR/anti-EGFR drug
Tarantino et al.	Retrospective, large database	1998-2009	23004	14789	19	NR	NR	HR 0.40<br>P<0.001	CT regimen not specified

Bajwa et al.	Retrospective, single center	1999-2005	32	35	NR	14.0	6.0	0.005	5-FU-based/Oxaliplatin/Irinotecan
Chan et al.	Retrospective, population-based cohort	2000-2002	286	125	24	14.0	6.0	<0.001	5-FU<br>Irinotecan
Yoon et al.	Prospective cohort study, single center	2000-2007	195	66	42	21	10	<0.001	FOLFOX/FOLFIRI/XELOX/XELIRI/ 5-FU-LV/capecitabine ± bevacizumab, cetuximab
Park et al.	Retrospective, single center	2000-2009	527	320	NR	21.4	14.1	<0.001	Oxaliplatin/Irinotecan/capecitabine ± bevacizumab/cetuximab
Ahn et al.	Retrospective, single center	2001-2009	28	36	25	12.43	3.58	<0.001	5-FU-LV/FOLFOX/FOLFIRI
Venderbosch et al.	Retrospective analysis of two RT	2003-2004<br>2005-2006	258<br>289	141<br>159	26<br>19	16.7<br>20.7	11.4<br>13.4	<0.0001<br><0.0001	Capecitabine + Oxaliplatin + Irinotecan<br>Capecitabine + Oxaliplatin+ Bevacizumab ± Cetuximab
Gulack et al.	Retrospective, large database	2003-2006	231	1215	40	9.2	7.6	<0.01	CT regimen not specified
Tanoue et al.	Retrospective, single center	2005-2009	38	36	47	30.6	20.8	0.0094	FOLFOX/FOLFIRI ± bevacizumab/cetuximab
Ichikawa et al.	Retrospective, single center	2005-2010	37	58	NR	30.7	21.9	0.031	CT regimen not specified
Gresham et al.	Retrospective, multicenter	2006-2008	378	139	24	17.9	7.9	<0.0001	FOLFOX/FOLFIRI ± bevacizumab/ capecitabine/5FU
Ahmed et al.	Retrospective, single center	2006-2010	313	256	33	Resection of PT + CT: 27	Only CT: 14	<0.001	FOLFOX/FOLFIRI ± bevacizumab/ cetuximab/panitumumab
Kim et al.	Retrospective, single center	2006-2010	72	252	30	17.2	13.6	0.002	Oxaliplatin-based or irinotecan-based ± anti-VEGF/anti-EGFR
‘t Lam-Boer et al.	Retrospective, large database	2008-2011	2746	3345	23	17.2	11.5	<0.001	CT regimen not specified
Wong et al.	Retrospective, large database	2009-2015	216	394	24	21	17	0.014	CT regimen not specified0 ± bevacizumab/cetuximab
Wang et al.	Prospective cohort study, single center	2011-2013	118	73	40	22.5	17.8	<0.01	FOLFOX/XELOX/FOLFIRI + bevacizumab

5-FU: 5-fluorouracil; BSC: best supportive care; CSS: cancer-specific survival; CT: chemotherapy; HR: hazard ratio; LV: leucovorin; PT: primary tumor; RT: randomized trial.

Xu H, Xia Z, Jia X, et al. Primary tumor resection is associated with improved survival in stage IV colorectal cancer: an instrumental variable analysis. Sci Rep. 2015;5:16516.

Konyalian VR, Rosing DK, Haukoos JS, et al. The role of primary tumour resection in patients with stage IV colorectal cancer. Colorectal Dis. 2007;9:430–437.

Ahmed S, Leis A, Fields A, et al. Survival impact of surgical resection of primary tumor in patients with stage IV colorectal cancer: results from a large population-based cohort study. Cancer. 2014;120(5):683-91.

Ahmed S, Fields A, Pahwa P, et al. Surgical Resection of Primary Tumor in Asymptomatic or Minimally Symptomatic Patients With Stage IV Colorectal Cancer: A Canadian Province Experience. Clin Colorectal Cancer. 2015;14(4):e41-7.

Beham A, Rentsch M, Püllmann K, et al. Survival benefit in patients after palliative resection vs non-resection colon cancer surgery. World J Gastroenterol. 2006;12:6634–6638.

Duraker N, Civelek Çaynak Z, et al. The impact of primary tumor resection on overall survival in patients with colorectal carcinoma and unresectable distant metastases: a prospective cohort study. Int J Surg. 2014;12:737–741.

Costi R, Mazzeo A, Di Mauro D, et al. Palliative resection of colorectal cancer: does it prolong survival? Ann Surg Oncol. 2007;14:2567–2576.

Stelzner S, Hellmich G, Koch R, et al. Factors predicting survival in stage IV colorectal carcinoma patients after palliative treatment: a multivariate analysis. J Surg Oncol. 2005;89:211–217.

Galizia G, Lieto E, Orditura M, et al. First-line chemotherapy vs. bowel tumor resection plus chemotherapy for patients with unresectable synchronous colorectal hepatic metastases. Arch Surg. 2008;143:352–8.

Ruo L, Gougoutas C, Paty PB, et al. Elective bowel resection for incurable stage IV colorectal cancer: prognostic variables for asymptomatic patients. J Am Coll Surg. 2003;196:722–8.

Mik M, Dziki L, Galbfach P, et al. Resection of the primary tumour or other palliative procedures in incurable stage IV colorectal cancer patients? Colorectal Dis. 2010;12:e61–e67.

Tsang WY, Ziogas A, Lin BS, et al. Role of primary tumor resection among chemotherapy-treated patients with synchronous stage IV colorectal cancer: a survival analysis. J Gastrointest Surg. 2014;18:592–598.

Ferrand F, Malka D, Bourredjem A, et al. Impact of primary tumour resection on survival of patients with colorectal cancer and synchronous metastases treated by chemotherapy: results from the multicenter, randomised trial Fédération Francophone de Cancérologie Digestive 9601. Eur J Cancer. 2013;49(1):90-7.

Ishihara S, Nishikawa T, Tanaka T, et al. Benefit of primary tumor resection in stage IV colorectal cancer with unresectable metastasis: a multicenter retrospective study using a propensity score analysis. Int J Colorectal Dis. 2015;30:807–812.

Faron M, Pignon JP, Malka D, et al. Is primary tumour resection associated with survival improvement in patients with colorectal cancer and unresectable synchronous metastases? A pooled analysis of individual data from four randomised trials. Eur J Cancer. 2015;51:166–176.

Kaufman MS, Radhakrishnan N, Roy R, et al. Influence of palliative surgical resection on overall survival in patients with advanced colorectal cancer: a retrospective single institutional study. Colorectal Dis. 2008;10:498–502.

Aslam MI, Kelkar A, Sharpe D, Jameson JS. Ten years experience of managing the primary tumours in patients with stage IV colorectal cancers. Int J Surg. 2010;8:305–313.

Karoui M, Roudot-Thoraval F, Mesli F, et al. Primary colectomy in patients with stage IV colon cancer and unresectable distant metastases improves overall survival: results of a multicentric study. Dis Colon Rectum. 2011;54:930–938.

Tarantino I, Warschkow R,Worni M, et al. Prognostic relevance of palliative primary tumor removal in 37,793 metastatic colorectal cancer patients: a population-based, propensity score-adjusted trend analysis. Ann Surg. 2015;262:112–120.

Bajwa A, Blunt N, Vyas S, et al. Primary tumour resection and survival in the palliative management of metastatic colorectal cancer. Eur J Surg Oncol. 2009;35:164–7.

Chan TW, Brown C, Ho CC, et al. Primary tumor resection in patients presenting with metastatic colorectal cancer: analysis of a provincial population-based cohort. Am J Clin Oncol. 2010;33:52–5.

Yoon YS, Kim CW, Lim SB, et al. Palliative surgery in patients with unresectable colorectal liver metastases: a propensity score matching analysis. J Surg Oncol. 2014;109: 239–244.

Park JH, Kim TY, Lee KH, et al. The beneficial effect of palliative resection in metastatic colorectal cancer. Br J Cancer. 2013;108:1425–1431.

Ahn HJ, Oh HS, Ahn Y, et al. Prognostic implications of primary tumor resection in stage IVB colorectal cancer in elderly patients. Ann Coloproctol. 2014;30:175–181.

Venderbosch S, de Wilt JH, Teerenstra S, et al. Prognostic value of resection of primary tumor in patients with stage IV colorectal cancer: retrospective analysis of two randomized studies and a review of the literature. Ann Surg Oncol. 2011; 18: 3252-60.

Gulack BC, Nussbaum DP, Keenan JE, et al. Surgical resection of the primary tumor in stage IV colorectal cancer without metastasectomy is associated with improved overall survival compared with chemotherapy/radiation therapy alone. Dis Colon Rectum. 2016;59:299–305.

Tanoue Y, Tanaka N, Nomura Y. Primary site resection is superior for incurable metastatic colorectal cancer. World J Gastroenterol. 2010;16:3561-6.

Ichikawa Y, Goto A, Kobayashi N, et al. Does resection of primary lesions show survival benefit for stage IV colorectal cancer patients with unresectable metastases? Hepatogastroenterology. 2013;60:1945–1949.

Gresham G, Renouf DJ, Chan M, et al. Association between palliative resection of the primary tumor and overall survival in a population-based cohort of metastatic colorectal cancer patients. Ann Surg Oncol. 2014;21:3917–3923.

Ahmed S, Leis A, Chandra-Kanthan S, et al. Surgical Management of the Primary Tumor in Stage IV Colorectal Cancer: A Confirmatory Retrospective Cohort Study. J Cancer. 2016;7(7):837-45.

Kim MS, Chung M, Ahn JB, et al. Clinical significance of primary tumor resection in colorectal cancer patients with synchronous unresectable metastasis. J Surg Oncol. 2014;110: 214–221.

't Lam-Boer J, Van der Geest LG, Verhoef C, et al. Palliative resection of the primary tumor is associated with improved overall survival in incurable stage IV colorectal cancer: A nationwide population-based propensity-score adjusted study in the Netherlands. Int J Cancer. 2016;139:2082-94.

Wong SF, Wong HL, Field KM, et al. Primary tumor resection and overall survival in patients with metastatic colorectal cancer treated with palliative intent. Clin Colorectal Cancer. 2016;15:e125-32.

Wang Z, Liang L, Yu Y, et al. Primary tumour resection could improve the survival of unresectable metastatic colorectal cancer patients receiving bevacizumab-containing chemotherapy. Cell Physiol Biochem. 2016;39:1239-46.
